# Feature and Score Fusion Based Multiple Classifier Selection for Iris Recognition

**DOI:** 10.1155/2014/380585

**Published:** 2014-07-10

**Authors:** Md. Rabiul Islam

**Affiliations:** Department of Computer Science & Engineering, Rajshahi University of Engineering & Technology, Rajshahi 6204, Bangladesh

## Abstract

The aim of this work is to propose a new feature and score fusion based iris recognition approach where voting method on Multiple Classifier Selection technique has been applied. Four Discrete Hidden Markov Model classifiers output, that is, left iris based unimodal system, right iris based unimodal system, left-right iris feature fusion based multimodal system, and left-right iris likelihood ratio score fusion based multimodal system, is combined using voting method to achieve the final recognition result. CASIA-IrisV4 database has been used to measure the performance of the proposed system with various dimensions. Experimental results show the versatility of the proposed system of four different classifiers with various dimensions. Finally, recognition accuracy of the proposed system has been compared with existing *N* hamming distance score fusion approach proposed by Ma et al., log-likelihood ratio score fusion approach proposed by Schmid et al., and single level feature fusion approach proposed by Hollingsworth et al.

## 1. Introduction

Biometrics deals with identification of individuals based on their biological or behavioral characteristics which provides the significant component of automatic person identification technology based on a unique feature like face, iris, retina, speech, palmprint, hand geometry, signature, fingerprint, and so forth [1]. Iris recognition technology is the most reliable existing biometric systems available because of the unique feature of human iris. Iris has some unique features such as accuracy, uniqueness, high information content, stability, reliability, and real-time access capability compared with other biometric patterns [2, 3]. Such unique feature in the anatomical structure of the iris facilitates the differentiation among individuals. The human iris pattern is not changeable and is constant over person's lifetime from one year of age until death [1, 4]. Iris is a thin circular diaphragm which is a part between the blackish pupil and the whitish sclera [5]. Because of this uniqueness and stability, iris recognition is a reliable person identification technique [6].

Though unimodal biometric system performs well in some of the cases, it involves a variety of problems like nonuniversality, susceptibility of spoofing, noises of sensed information, intraclass variations, and interclass similarities [7]. Multimodal biometric system (i.e., the combination of more than one unimodal biometric system) can solve some of the above-mentioned problems. Recently, scientists are attempting to combine multiple modalities for person identification which are referred to as multimodal biometrics. Multimodal biometric identification systems are capable of utilizing more than one physical, behavioral, or chemical characteristic for enrollment. Multimodal biometric systems consolidate the evidence presented by multiple biometric sources and typically provide better recognition performance compared to systems based on a single biometric modality. For combining the multimodal information, generally four different fusion strategies, that is, sensor level fusion, feature level fusion, score level fusion, and decision level fusion, have been used for various multimodal systems [8, 9]. In sensor level fusion, information is taken from multiple sensors that can be processed and integrated to generate a new vector. Extracted features are fused to produce a combined vector in feature level fusion. For score level fusion, scores are counted from different classifiers and combined to get the final result. Lastly, several classifiers output is combined to achieve the multimodal biometric system in decision fusion approach which includes Multiple Classifier Selection (MCS) and Dynamic Classifier Selection (DCS).

In this paper, a new hybrid fusion based pair of iris recognition approaches is proposed where three different fusion levels such as feature fusion, score fusion, and decision fusion are used. Discrete Hidden Markov Model (DHMM) has been used to classify the input iris pattern. For decision fusion, four different classifiers output, that is, left iris based unimodal DHMM classifier, right iris based unimodal DHMM classifier, left-right iris feature fusion based multimodal DHMM classifier, and left-right iris log-likelihood ratio score fusion based multimodal DHMM classifier, is combined to achieve the final result. The next sections of the paper deal with related work and proposed fusion scheme, automated iris segmentation and feature extraction techniques, and experimental results of each of the four different classifiers and their combined output with the comparison of existing multimodal iris recognition system.

## 2. Literature Review and Architecture of the Proposed System

A very little amount of work has been done in iris recognition where multiple modalities of iris have been used. Most of the multimodal iris biometric work combines iris with other biometric techniques. The largest cluster of papers in this area deals with the combination of face and iris and another multibiometric system involves almost any combination of iris and some other multimodalities like fingerprint, palmprint, speech, and so forth [11]. Two different fusion strategies that is, feature and score fusion have been used in those works. Researchers used these fusion techniques with different strategies in the areas of multimodal techniques. In feature fusion, person identification has performed by combining iris and facial feature vector [12]. Wang et al. also used face and iris common feature vector for multimodal biometric recognition [13, 14]. Rattani and Tistarelli proposed a multiunit multimodal biometric system where face, left iris and right iris image vector were used and fused these three different features to enhance the performance [15]. Iris and fingerprint multimodal feature fusion based cryptographic key generation system [16] and person identification system [17] were developed. Various multimodal score level fusion schemes were also proposed by different researchers. In [18], different algorithms were used to calculate the score of iris and facial multimodalities and Support Vector Machine (SVM) was used to combine those scores. User specific score fusion approach of iris and face [19], iris and fingerprint based multimodal score fusion [20], score fusion of iris, and palmprint multibiometric system [21] were also developed.

Some of the fusion approaches were proposed for iris recognition where also feature and score fusion methods were used. By using iris feature fusion method, Vatsa et al. combined left and right iris image for multimodal biometric system [22]. Hollingsworth et al. [23] created a single average image from multiple frames from iris video which showed that single level fusion is better than multigallery score fusion method. In score fusion method, Ma et al. [24] used three different templates from an iris image and averaged their scores to get the final result. Minimum match score was used instead of iris image averaging to collect the final score for iris biometric recognition by Krichen et al. [25]. Schmid et al. developed log-likelihood ratio method to fuse the score which can better perform than Hamming distance based score fusion [26].

Hollingsworth et al. [27] proposed an approach of image averaging using single level feature from iris image to improve the matching performance. They compared their system with three reimplemented score fusion approaches of Ma et al. [24], Krichen et al. [25], and Schmid et al. [26]. They reported that the proposed feature fusion approach performed better than Ma's or Krichen's score level fusion methods of *N* Hamming distance scores and also performed better than Schmid's log-likelihood method of score fusion.

**Figure 1:**
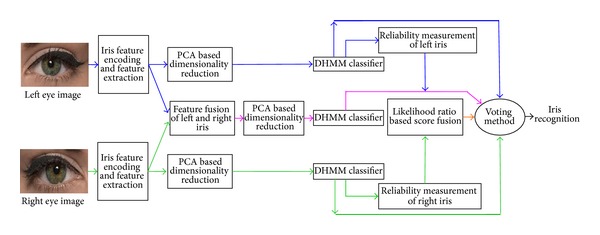
Block diagram of the proposed feature and decision fusion based Multiple Classifier Selection for iris recognition.

In this proposed work, a combined approach of feature fusion and score fusion based pair of iris multibiometric systems has developed which is shown in Figure 1. Features from left and right iris images are fused and log-likelihood ratio based score fusion method is applied to find the score of iris recognition. Finally, to take the final recognition result, voting method is applied to combine the output of Multiple Classifier Selection (MCS) such as the individual modality of each iris (i.e., left and right iris), feature fusion based modality, and log-likelihood ratio based modality. Discrete Hidden Markov Model (DHMM) has been used as a classifier and Principal Component Analysis (PCA) has been applied to reduce the dimensionality of the iris feature in different levels of the overall proposed approach. Various experiments have been performed on the proposed feature and decision fusion Based MCS approach with existing hamming distance score fusion approach proposed by Ma et al. [24], log-likelihood ratio score fusion approach proposed by Schmid et al. [26], and feature fusion approach proposed by Hollingsworth et al. [27].

## 3. Iris Segmentation and Feature Extraction

Since iris image preprocessing and feature extraction play a vital role of the overall recognition performance, standard iris localization, segmentation, normalization, and feature encoding procedure have been applied in the proposed system.

**Figure 2:**
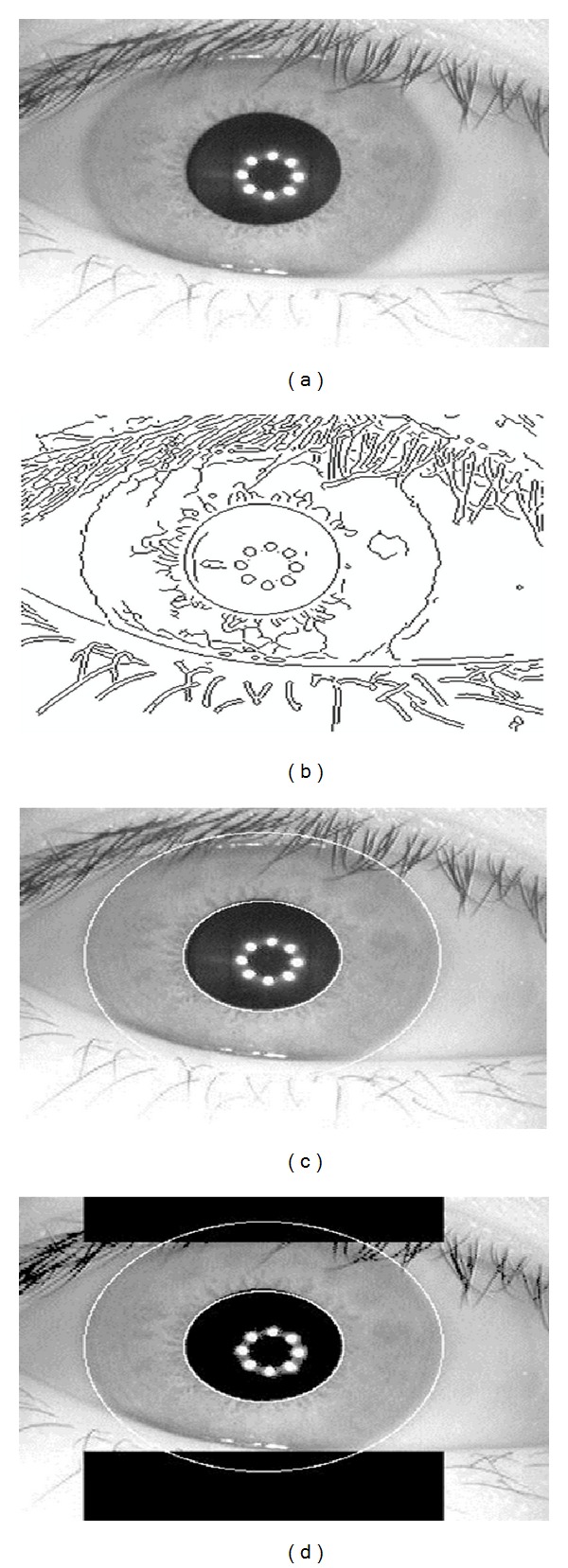
(a) Eye image, (b) edge map using canny edge detector, (c) results of circular Hough transform, and (d) removing the eyelids part.

The first step is to isolate the actual iris region from the eye image. For this, an edge map is created using edge detection algorithm with canny directional edge detector.  Figure 2(b) shows the edge map after applying canny edge detector. To extract the original iris region, two different boundaries have to be predicted. As a result, a vertical edge map has been created for detecting the outer boundary, that is, iris/sclera boundary, and to observe the inner boundary, that is, pupil/iris boundary, a horizontal edge map has been created. Circular Hough transform has been applied to deduce the center coordinates and radius for the outer and inner boundaries of the iris [28, 29]. The results of using circular Hough transform are shown in Figure 2(c). After doing that, we have to remove those parts from the iris, which are overlapped by eyelids. Eyelids were isolated by first fitting a line to the upper and lower eyelid using the linear Hough transform [30]. A second horizontal line is then drawn, which intersects with the first line at the iris edge that is closest to the pupil. This process is done for both the top and bottom eyelids. The second horizontal line allows maximum isolation of eyelid regions. The result of removing the eyelids region is shown in Figure 2(d).

**Figure 3:**
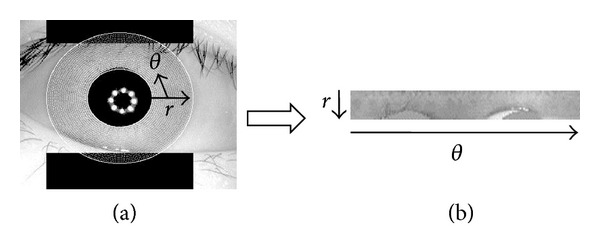
(a) Normalization process and (b) iris region with fixed dimension.

In normalization, iris region is transformed into a fixed dimension so that one can compare two different iris images with the same spatial location. Dimension inconsistencies of the iris region occur due to stretching of iris caused by pupil dilation for varying levels of light illumination, image capturing distance, angular deflection of camera and eye, and so forth. Normalization process removes all of the above-mentioned difficulties to produce a fixed size iris vector. Daugman's Rubber Sheet Model [31] has been used to normalize the iris region. In this process, each point of the iris region converted into a pair of polar coordinates (*r*, *θ*), where *r* is on the interval between 0 and 1 and *θ* is the angle between 0 and 2*π* which is shown in Figure 3. Problem can occur for rotation of the eye within the eye socket in iris image. For this reason, the center of the pupil has been considered as the reference point and a number of data points are selected along each radial line which is called the radial resolution. In this way, we can get the fixed dimension of iris region which is shown in Figure 3(b).

**Figure 4:**
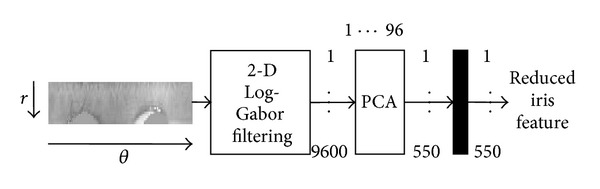
Feature encoding and dimensionality reduction process.

For extracting the feature, it is important to extract the most important information presented in the iris region. There are different alternative techniques that can be used for feature extraction which includes Gabor filtering techniques, Zero-crossing 1D wavelet filters, Log-Gabor filters, and Haar wavelet. In this work, Log-Gabor filtering technique has been applied to extract the iris features effectively. 9600 feature values have been taken from each iris region. Principal Component Analysis method has been used to reduce the dimension of the feature vector where 550 feature values have been taken. Feature extraction and dimensionality reduction process are shown in Figure 4.

## 4. Iris Recognition Using HMM Classifier

In training phase of the proposed iris recognition system, for each iris *k*, DHMM (Discrete HMM), *θ*_*k*_ has been built [32]. The model parameters (*A*, *B*, *θ*) have been estimated to optimize the likelihood of the training set observation vector for the *k*th iris by using Baum-Welch algorithm. The Baum-Welch reestimation formula has been considered as follows [33]:

(1) $$\begin{array}{c} {\overset{-}{\Pi }}_{i}=\gamma_{1}\left(i\right),\\ {\bar{a}}_{ij}=\frac{\sum_{t=1}^{T-1}\xi_{t}\left(i,j\right)}{\sum_{t=1}^{T-1}\gamma_{t}\left(i\right)},\\ {\bar{b}}_{j}\left(\vec{k}\right)=\frac{\sum_{t=1(s,t,{\vec{o}}_{t}={\vec{v}}_{k})}^{T}\gamma_{t}\left(j\right)}{\sum_{t=1}^{T}\gamma_{t}\left(j\right)}, \end{array}$$

where

(2) $$\begin{array}{c} \xi_{t}\left(i,j\right)=\frac{\alpha_{t}\left(i\right)a_{ij}b_{j}\left({\bar{o}}_{t+1}\right)\beta_{t+1}\left(j\right)}{\sum_{i=1}^{N}\sum_{j=1}^{N}\alpha_{t}\left(i\right)a_{ij}b_{j}\left({\bar{o}}_{t+1}\right)\beta_{t+1}\left(j\right)},\\ \gamma_{t}\left(i\right)=\overset{N}{\underset{j=1}{\sum }}\xi_{t}\left(i,j\right). \end{array}$$

**Figure 5:**
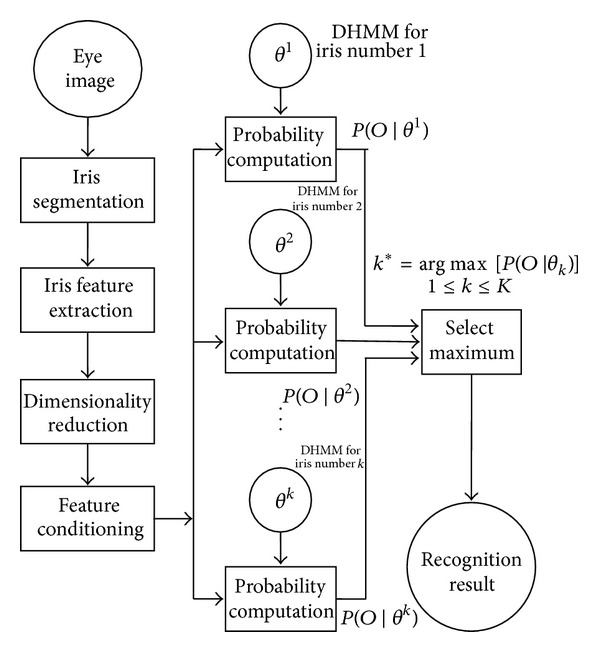
Block diagram of DHMM based iris recognition system (modified) [10].

In the testing phase, for each unknown iris to be recognized, the processing shown in Figure 5 has been carried out. This procedure includes

(i) measurement of the observation sequence, *O* = {*o*_1_, *o*_2_,…, *o*_*n*_}, via a feature analysis of the iris pattern,
(ii) transforming the continuous values of *O* into integer values,
(iii) calculation of model likelihoods for all possible models, *P*(*O*∣*θ*_*k*_), 1 ≤ *k* ≤ *K*,
(iv) declaration of the iris as k* person whose model likelihood is the highest—that is,
  (3) $$\begin{array}{c} k^{*}=\underset{1\leq k\leq K}{\arg\;\max}\left[P\left(O\;|\;\theta_{k}\right)\right]. \end{array}$$

In this proposed work, the probability computation step has been performed using Baum's Forward-Backward algorithm [33, 34].

Medium sized iris dataset has been used for most of the recent iris recognition tasks. However it is difficult to evaluate the performance accurately with this problem. However, more and more large-scale iris recognition systems are deployed in real-world applications. Many new problems are met in classification and indexing of large-scale iris image databases. So scalability is another challenging issue in iris recognition. CASIA-IrisV4 iris database has been released to promote research on long-range and large-scale iris recognition systems. As a result to measure the performance of the overall iris recognition system, CASIA-IrisV4 iris database [35] has been used.

CASIA-IrisV4 database was developed and released to the international biometrics community and updated from CASIA-IrisV1 to CASIA-IrisV3 since 2002. More than 3,000 users from 70 countries or regions have downloaded CASIA-Iris and much excellent work on iris recognition has been done based on these iris image databases. CASIA-IrisV4 contains 54601 iris images where 1800 are genuine and 1000 are virtual subjects. Iris images are collected under near infrared illumination or synthesized and represented as 8-bit gray level. There are six data sets which were collected at different times and four different methodologies were used, that is, CASIA-Iris-Interval, CASIA-Iris-Lamp, CASIA-Iris-Distance, and CASIA-Iris-Thousand. Since CASIA-Iris-Interval is well suited for the detailed texture features of iris images which are captured by close-up iris camera, this dataset has been used for the experimental work of the proposed system. The most compelling feature of this database is to design a circular NIR LED array with suitable luminous flux for iris imaging.

**Figure 6:**
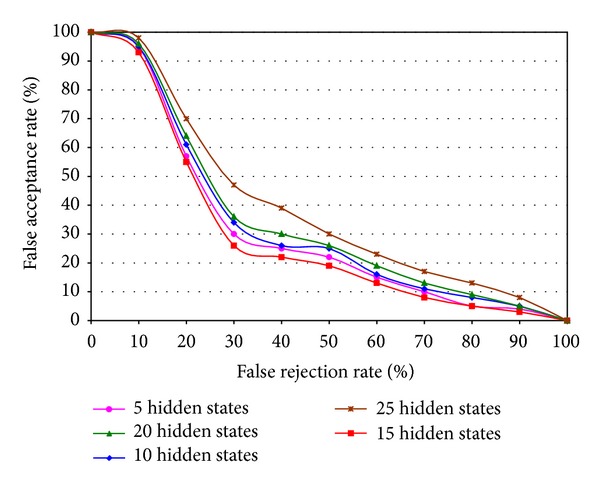
ROC curve of left iris and right iris based unimodal system.

For measuring the accuracy of individual left iris and right iris based unimodal recognition system, the critical paramerter, that is, the number of hidden states of DHMM, can affect the performance of the system. A tradeoff is made to explore the optimum value of the number of hidden states and comparison results with Receiver Operating Characteristics (ROC) curve are shown in Figure 6 which represents the left and right iris based unimodal recognition performance combinedly.

## 5. Feature Fusion Based HMM Classifier

Feature level fusion of iris recognition can significantly improve the performance of a multibiometric system besides improving population coverage, deterring spoof attacks, increasing the degrees-of-freedom, and reducing the failure-to-enroll rate. Although the storage requirements, processing time, and the computational demands of feature fusion based system are much higher than unimodal system for iris recognition [36], effective integration of left and right iris features can remove or reduce most of the above-mentioned problems. Feature level fusion of left and right iris features is an important fusion strategy which can improve overall system performance of the proposed system. In feature level fusion, sufficient information can exist compared with score level fusion and decision level fusion. As a result, it can be expected that feature level fusion can achieve greater performance over other fusion strategies of the proposed multimodal iris recognition system.

**Figure 7:**
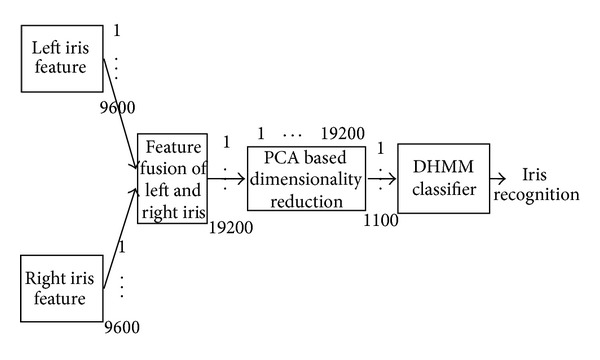
Feature fusion based multimodal iris recognition system.

Feature level fusion can be found by simple concatenation of the feature sets taken from left and right information source. By concatenating two feature vectors, *U*′ = {*u*_1_′, *u*_2_′,…, *u*_*m*_′} and *V*′ = {*v*_1_′, *v*_2_′,…, *v*_*n*_′}, a new feature vector, *UV*′ = {*u*_1_′, *u*_2_′,…, *u*_*m*_′, *v*_1_′, *v*_2_′,…, *v*_*n*_′},  *UV* ∈ *R* ^*m*+*n*^, has been created. The objective is to combine these two feature sets in order to create a new feature vector, *UV*, that would better represent the individual. To combine left and right iris feature vectors, the dimensionality of new feature vector is very large. As a result, dimensionality reduction technique is necessary to reduce the searching domain of learned database. The feature selection process chooses a minimal feature set of *k*, where *k* < (*m* + *n*) that improves the performance on a trained set of feature vectors. The objective of this phase is to find out optimal subset of features from the complete feature set. Principal Component Analysis [37] has been used to reduce the dimensionality of the feature vector.  Figure 7 shows the process of left and right iris feature fusion based multimodal technique of the proposed system.

**Figure 8:**
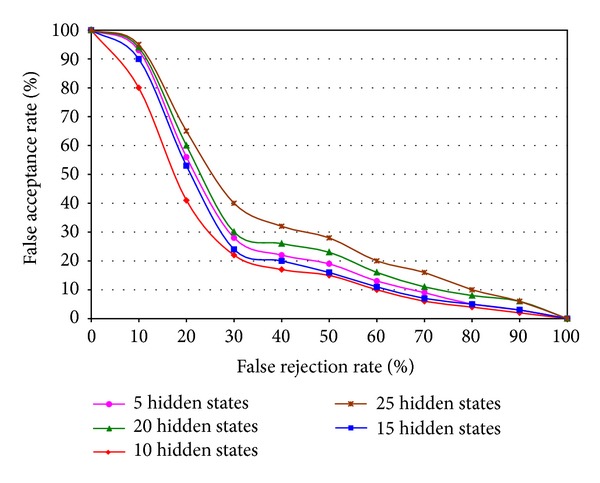
Recognition performance of left-right iris feature fusion based multimodal system among different number of hidden states of DHMM.

**Figure 9:**
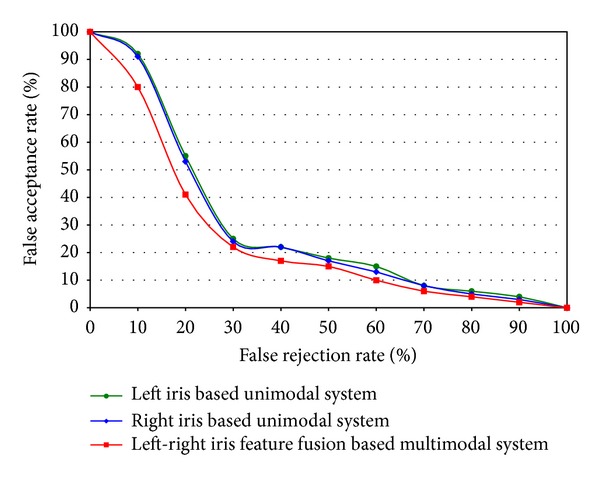
Performance comparison among unimodal left iris, unimodal right iris, and left-right iris feature fusion based multimodal recognition system.

In feature level fusion, optimum value of the number of hidden states of DHMM has been chosen and Figure 8 shows the comparison results with ROC curve.  Figure 9 shows the performance comparison among unimodal left iris, unimodal right iris, and left-right iris feature fusion based recognition. Since the primary goal of left-right iris feature fusion based multimodal iris recognition system is to achieve the performance which is equal to or better than the performance of any left or right unimodal iris recognition system. When the noise level is high of right iris, the left iris unimodal system performs better than the right iris unimodality; thus the left-right iris recognition performance should be at least as good as that of the right iris unimodal system. When the noise level is high of left iris, the right iris recognition performance is better than the left one and the integrated performance should be at least the same as or better than the performance of the right iris recognition. The system also works very well when left and right iris image do not contain noises.  Figure 9 shows the above-mentioned performance of the left-right feature fusion based multimodal iris recognition.

## 6. Likelihood Ratio Score Fusion Based HMM Classifier

In left-right iris feature fusion based method, the left iris and right iris features from all modalities are combined into one high dimensional vector. But the method considers all modalities with equal weight and it is the main disadvantage of the feature fusion based multimodal method. This problem can be solved by using different weights according to different noise conditions of the left and right iris modalities. The method of score fusion by assigning weights of each modality can be used for this purpose. This also allows the dynamic adjustment of the importance of each stream through the weights according to its estimated reliability.

**Figure 10:**
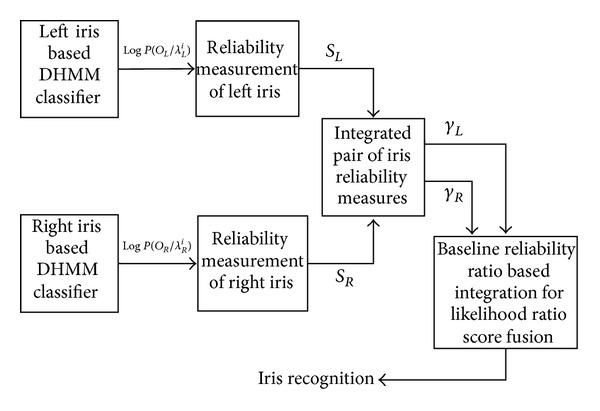
Process of likelihood ratio score fusion based pair of iris recognition systems.

Left-right iris likelihood ratio based score fusion method is a score fusion technique where the reliability of each modality is measured by using the output of DHMM classifier for both the left and right iris features. If one of the modalities becomes corrupted by noise, the other modality filling the gap by making its weight and the recognition rate will increase to use this score fusion based method. This is also valid in the case of a complete interruption of one stream. In this case, the corrupted modality should be close to maximum and the weight assigned to the missing stream close to zero. This practically makes the system revert to single stream recognition automatically. This process is instantaneous and also reversible; that is, if the missing stream is restored, the modality would decrease and the weight would increase to the level before the interruption. The integrated weight which determines the amount of contribution from each modality in left-right iris score fusion based recognition system is calculated from the relative reliability of the two modalities.  Figure 10 shows the process of using likelihood ratio score fusion based pair of iris recognition systems.

To calculate the score fusion result, the DHMM outputs of individual iris (i.e., left and right) recognition are combined by a weighted sum rule to produce the final score fusion result. For a given left-right iris test datum of *O*_*L*_ and *O*_*R*_, the recognition utterance C* is given by [38]

(4) C∗=arg max⁡i{γlog⁡P(OLλLi)+(1−γ)log⁡P(ORλRi)},

where *λ*_*L*_^*i*^ and *λ*_*R*_^*i*^ are the left iris and the right iris DHMMs for the *i*th utterance class, respectively, and log⁡*P*(*O*_*L*_/*λ*_*L*_^*i*^) and log⁡*P*(*O*_*R*_/*λ*_*R*_^*i*^) are their log-likelihood against the *i*th class.

The weighting factor *γ* determines the contribution of each modality for the final decision. From the two most popular integration approaches such as baseline reliability ratio based integration and *N*-best recognition hypothesis reliability ratio based integration, baseline reliability ratio based integration has been used where the integration weight is calculated from the reliability of each individual modality. The reliability of each modality can be calculated by the most appropriate and best in performance as [39]

(5) $$\begin{array}{c} S_{m}=\frac{1}{N-1}\overset{N}{\underset{i=1}{\sum }}\left(\underset{j}{\max}\log P\left(\frac{O}{\lambda^{j}}\right)-\log P\left(\frac{O}{\lambda^{i}}\right)\right), \end{array}$$

which means the average difference between the maximum log-likelihood and the other ones are used to determine the reliability of each modality. *N* is the number of classes being considered to measure the reliability of each modality, *m* ∈ {*L*, *R*}.

Then the integration weight of left iris reliability measure *γ*_*L*_ can be calculated by [40]

(6) $$\begin{array}{c} \gamma_{L}=\frac{S_{L}}{S_{L}+S_{R}}, \end{array}$$

where *S*_*L*_ and *S*_*R*_ are the reliability measure of the outputs of the left iris and right iris DHMMs, respectively.

The integration weight of right iris modality measure can be found as

(7) $$\begin{array}{c} \gamma_{R}=\left(1-\gamma_{L}\right). \end{array}$$

**Figure 11:**
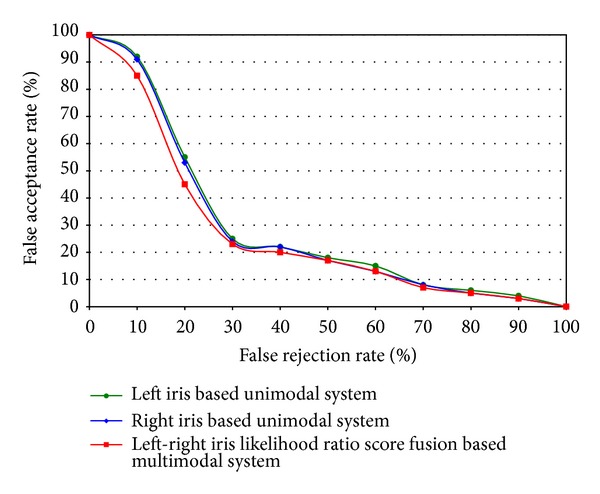
Results of comparison among unimodal left iris, unimodal right iris, and multimodal left-right iris recognition system.

The results after applying score fusion approach for iris recognition system are shown in Figure 11. The results show the comparison among unimodal left iris, unimodal right iris, and left-right iris score fusion based multimodal recognition system. Here, the score fusion approach achieves higher recognition rate than any individual unimodal system of left and right iris.

## 7. Multiple Classifier Fusion for the Proposed System

An effective way to combine multiple classifiers is required when a set of classifiers outputs are created. Various architectures and schemes have been proposed for combining multiple classifiers [41]. The majority vote [42–45] is the most popular approach. Other voting schemes include the maximum, minimum, median, nash [46], average [47], and product [48] schemes. Other approaches to combine classifiers include the rank-based methods such as the Borda count [49], the Bayes approach [44, 45], the Dempster-Shafer theory [45], the fuzzy integral [50], fuzzy connectives [51], fuzzy templates [52], probabilistic schemes [53], and combination by neural networks [54]. Majority, average, maximum, and nash voting techniques [41, 47] have been used to find out the most efficient voting technique for combining four classifiers output in this work.

In majority voting technique, the correct class is the one most often chosen by different classifiers. If all the classifiers indicate different classes or in the case of a tie then the one with the highest overall output is selected to be the correct class. For maximum voting technique, the class with the highest overall output is selected as the correct class,

(8) $$\begin{array}{c} Q\left(x\right)=\overset{K}{\underset{i=1}{\arg\;\max}}y_{i}\left(x\right), \end{array}$$

where *K* is the number of classifiers and *y*_*i*_(*x*) represents the output of the *i*th classifier for the input vector *x*.

Averaging voting technique averages the individual classifier outputs confidence for each class across all of the ensemble. The class output yielding the highest average value is chosen to be the correct class,

(9) $$\begin{array}{c} Q\left(x\right)=\overset{N}{\underset{j=1}{\arg\;\max}}\left(\frac{1}{K}\overset{K}{\underset{i=1}{\sum }}y_{ij}\left(x\right)\right), \end{array}$$

where *N* is the number of classes and *y*_*ij*_(*x*) represents the output confidence of the *i*th classifier for the *j*th class for the input *x*.

In nash voting technique, each voter assigns a number between zero and one for each candidate and then compares the product of the voter's values for all the candidates. The highest is the winner:

(10) $$\begin{array}{c} Q\left(x\right)=\overset{N}{\underset{j=1}{\arg\;\max}}\overset{K}{\underset{i=1}{\prod }}y_{ij}. \end{array}$$

**Figure 12:**
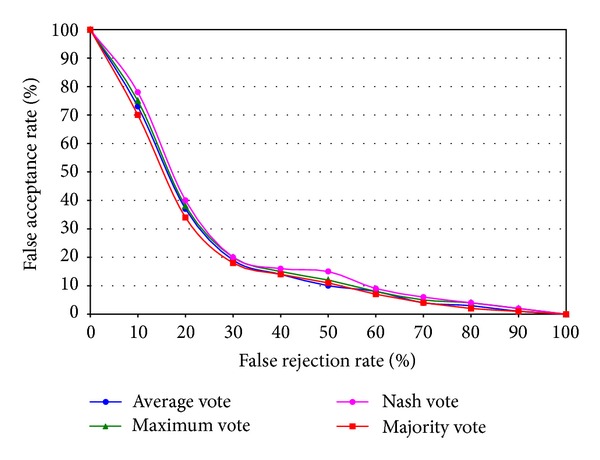
Results of different types of voting techniques for the proposed system.

Different types of voting techniques, that is, average vote, maximum vote, nash vote, and majority vote, have been applied to measure the accuracy of the proposed system.  Figure 12 shows the comparison results of different types of voting techniques. Though the recognition rates are very close to each other of the voting techniques, majority voting technique gives the highest recognition rate of the proposed system.

**Figure 13:**
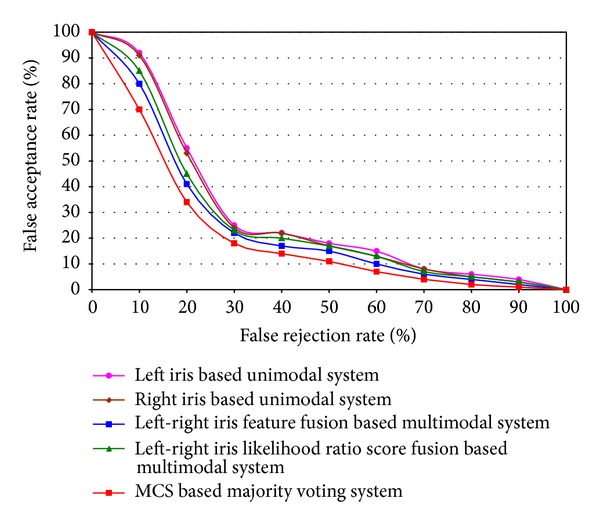
Performance comparison among each unimodal, multimodal, and MCS based majority voting technique for iris recognition.

The results of each unimodal system of iris, left-right iris feature fusion based multimodal system, likelihood ratio based score fusion based multimodal system, and MCS based majority voting technique are compared, which is shown in Figure 13. From the result, it has been shown that MCS based majority voting technique achieves higher performance than any other existing approach of iris recognition system.

## 8. Performance Analysis of the Proposed System

**Figure 14:**
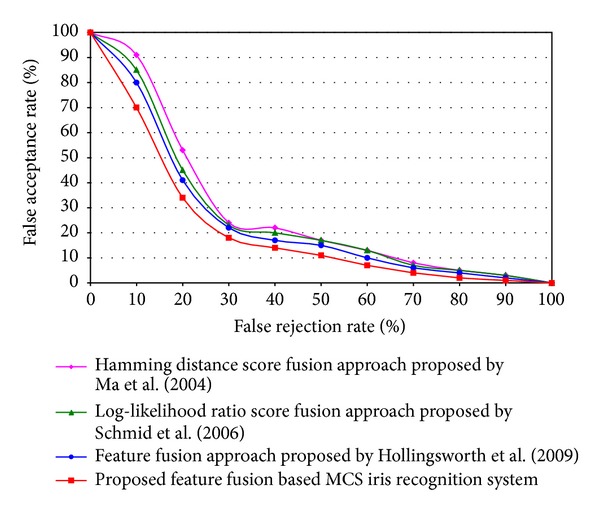
Performance comparison between proposed and different existing approaches of multimodal iris recognition.

The proposed approach of feature and score fusion based Multiple Classifier Selection (MCS) performance has been compared with existing hamming distance score fusion approach proposed by Ma et al. [24], log-likelihood ratio score fusion approach proposed by Schmid et al. [26], and feature fusion approach proposed by Hollingsworth et al. [27].  Figure 14 shows the results where the proposed system has achieved the highest recognition rate over all of the above-mentioned existing iris recognition system. Hamming distance score fusion approach proposed by Ma et al. has been rebuilt for measuring the performance comparison with the proposed approach. From the ROC curve, it is shown that existing feature fusion approach of Hollingsworth et al. [27] approach gives higher recognition result compared with hamming distance score fusion approach of Ma et al. [24] and log-likelihood ratio score fusion approach of Schmid et al. [26]. Finally, the proposed feature and decision fusion based MCS system performs all over the existing multimodal such as any feature fusion and any score fusion approach. The reason is that the existing approaches applied only either feature fusion or score fusion technique. But in this proposed approach, feature fusion and score fusion techniques are combined with individual left iris and right iris recognition technique. These four different classifiers output (i.e., unimodal left iris recognition classifier, unimodal right iris recognition classifier, left-right iris feature fusion based classifier, and left-right iris likelihood ratio score fusion based classifier) is combined using Multiple Classifier Selection (MCS) through majority voting technique. Since four classifiers are used as the input for the majority voting technique, there is a chance for a tie. In that case, since left-right iris feature fusion based multimodal system can achieve higher performance than any other unimodal and likelihood ratio score fusion based multimodal system which is shown in Figure 12, the output of left-right iris feature fusion based multimodal system output has been taken to break the tie. Two unimodal systems are used here because if one unimodal system fails to recognize then the other unimodal system retains the accurate output as an associate with each other. Since the feature set contains richer information about the raw biometric data than the final decision, integration at feature level fusion is expected to provide better recognition results. As a result, left and right iris feature fusion have applied to improve the performance of the proposed system. In feature fusion, the features for both left and right iris modalities are integrated with equal weights but decision of different classifiers can be fused with different weights according to the noise level of left and right iris. Likelihood ratio score fusion based iris recognition system has been applied in this proposal to combine the classifier output nonequally. When these four different classifiers outputs are combined with MCS based majority voting technique, the proposed multimodal system takes all of the above-mentioned advantages which gives the highest recognition rate than other existing approaches of Ma et al., Schmid et al., and Hollingsworth et al. proposed multimodal iris recognition.

The time required to fuse the output of the classifiers is directly proportional to the number of modalities used for the proposed system. Since four different classifiers are used in this system, the learning and testing time will increase. The execution time of the proposed system is increased enough with the uses of the number of classifiers for majority voting method. Reduced processing time of the proposed system might be the further work of this system such that the proposed system can work like real-time environment and large population supported applications.

## 9. Conclusions and Observations

Experimental results show the superiority of the proposed multimodal feature and score fusion based MCS system over existing multimodal iris recognition systems proposed by Ma et al., Schmid et al., and Hollingsworth et al. Though CASIA-IrisV4 dataset has been used for measuring the performance of the proposed system, the database has some limitations. CASIA iris database does not contain specular reflections due to the use of near infrared light for illumination. However, some other iris databases such as LEI, UBIRIS, and ICE contain the iris images with specular reflections and few noise factors, which are caused by imaging under different natural lighting environments. The proposed system can be tested on the above-mentioned databases to measure the performance with natural lighting conditions at various noise levels. Since the proposed system can work for the offline environment, the execution time can be reduced so that it can work for real-time applications.
